# Does Longer Leukocyte Telomere Length and Higher Physical Fitness Protect Master Athletes From Consequences of Coronavirus (SARS-CoV-2) Infection?

**DOI:** 10.3389/fspor.2020.00087

**Published:** 2020-06-16

**Authors:** Herbert Gustavo Simões, Thiago Santos Rosa, Caio Victor Sousa, Samuel da Silva Aguiar, Daisy Motta-Santos, Hans Degens, Marko T. Korhonen, Carmen Silvia Grubert Campbell

**Affiliations:** ^1^Graduate Program in Physical Education, Catholic University of Brasilia, Brasília, Brazil; ^2^Bouve College of Health Sciences, Northeastern University, Boston, MA, United States; ^3^Department of Physical Education, University Center UDF, Brasilia, Brazil; ^4^School of Physical Education, Physiotherapy, and Occupational Therapy, UFMG, Belo Horizonte, Brazil; ^5^Department of Sciences, Manchester Metropolitan University, Manchester, United Kingdom; ^6^Institute of Sport Science and Innovations, Lithuanian Sports University, Kaunas, Lithuania; ^7^Gerontology Research Center, Faculty of Sport and Health Sciences, University of Jyväskylä, Jyväskylä, Finland

**Keywords:** aging, immune system, older athlete, telomere attrition, COVID-19, quarantine, coronavirus

## The SARS-CoV-2 Infection and Immune System

We are waging a war against a deadly virus that has already resulted in the death of thousands of people worldwide (Burn-Murdoch, 2020). Against this invisible enemy, we have a sophisticated defense: our immune system. After infection, leukocytes proliferate and send signals to other immune cells to replicate and differentiate and, hence, increasing recruits to the army to combat the invaders in an attempt to ensure survival (Immunology, 2013).

## Leukocyte Telomere Length, Immunosenescence, and Survival From Infections: The Power of Physical Exercise

During every cell division, telomeres shorten and this shortening will over time result in dysfunctional proteins and cells, leading to apoptosis, cell senescence, and ultimately death (Arbeev et al., 2020). This shortening is accelerated during chronic inflammation and oxidative stress, as they stimulate cell division for tissue repair and the immunological response (Blackburn et al., 2015). Shortened leucocyte telomeres, a marker of immunosenescence, may hamper the effectiveness of these cells to replicate and contribute to the diminished resistance to infections often seen in older individuals, particularly if their immune system is already challenged by chronic diseases, systemic inflammation, or other morbidities (Castelo-Branco and Soveral, 2014). Given the above observations, it is not surprising that most deaths caused by COVID-19 occur among frail sedentary elderly people with comorbidities (Abduljalil and Abduljalil, 2020).

Lymphopenia, characterized by a low number of CD4+ and CD8+ T lymphocytes, may well-contribute to the poor prognosis in more severe cases of COVID-19 (Tan et al., 2020), and may be the consequence of replicative failure and early lymphocyte senescence. This then supports the importance of telomerase activity and long leukocyte telomeres for immune homeostasis and a better outcome while facing infections (Helby et al., 2017). It is thus not surprising that longer leukocyte telomeres are indeed associated with better survival from sepsis and a lower severity of acute respiratory syndrome in critically ill patients (Liu et al., 2020).

**Figure 1:**
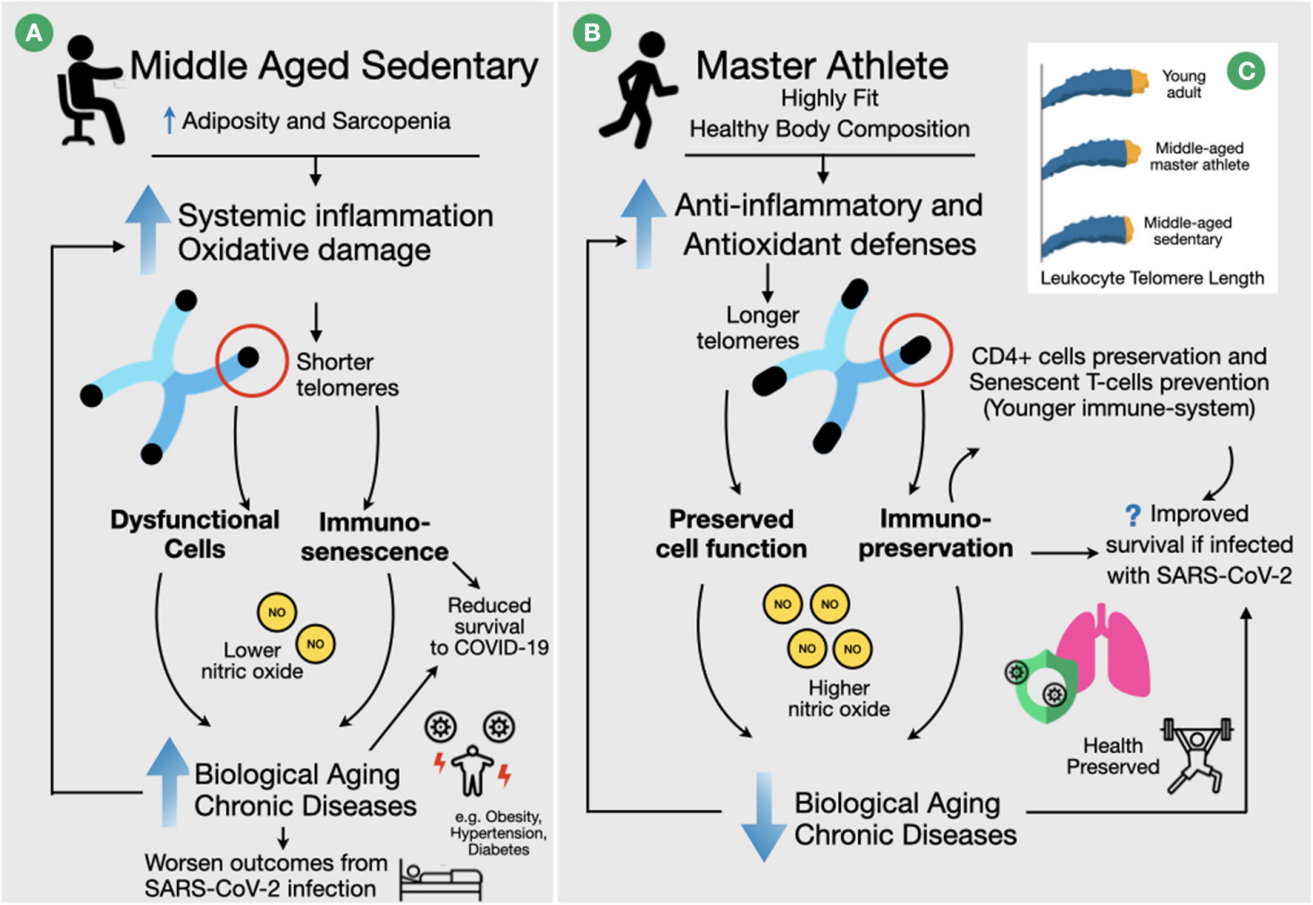
Physical fitness and age-related biomarkers in regards to immune function, and possible outcomes in case of SARS-CoV-2 infection. **(A)** For sedentary person telomere length decreases during aging mainly due to associated chronic inflammation (“inflammaging”) and oxidative stress. Shorter telomere lengths lead to dysfunctional cells and immunosenescense that in turn contribute to a higher incidence of chronic diseases and immunosuppression. These conditions lead to a worsened prognosis in case of SARS-CoV-2 infection. **(B)** On the other hand, well-conditioned Master Athletes have a better anti-inflammatory profile and improved anti-oxidant defenses that are associated with longer leukocyte telomere lengths, preserved cellular function, immunosenescence prevention, and increased levels of nitric oxide compared to sedentary peers. These positive adaptations reduce the risk of developing chronic diseases and, if infected by SARSCoV-2, Master Athletes will possibly have a better outcome while facing COVID-19. **(C)** Illustrative representation of telomere length in leukocytes of young adults, middle-aged master athletes and sedentary peers. According to recent studies, the telomere length of master athletes is greater than that of age-matched sedentary, and may not differ from young adults, suggesting that master athletes are biologically younger than their chronological age (Simoes et al., 2017; Aguiar et al., 2019; Sousa et al., 2019).

In this context it is interesting to note that older individuals engaged in high levels of physical activity (Puterman et al., 2010; Sjogren et al., 2014), have longer leukocyte telomeres and are biologically younger and healthier than age-matched sedentary older people (i.e., better blood pressure, autonomic balance, body composition, and lipid profile; Simoes et al., 2017; Deus et al., 2019; Sousa et al., 2019). They also have a preserved proportion of naive CD4+ cells without senescent T-cells accumulation than age-matched sedentary people (Minuzzi et al., 2018). This may at least partly be due to their lower level of systemic inflammation and oxidative damage (Aguiar et al., 2019, 2020). It is to be expected that such a profile will significantly enhance the ability of master athletes to combat infections, including COVID-19, above that seen in their unfit age-matched peers (Figure 1). Taken together, the increased antioxidant defenses and anti-inflammatory cytokines lead to an attenuated telomere attrition over life and attenuate biological aging, including that of the immune system (Figure 1), and will preserve immune homeostasis and proper lymphocyte replication (Pedersen and Toft, 2000; Bopp et al., 2004), possibly preventing COVID-induced lymphopenia, in master athletes.

## Nitric Oxide, Leukocyte Telomere Length, and the Biological Aging of Master Athletes

Master athletes are individuals who continue to train and compete in sporting events beyond middle age. Currently, it is not rare to see octogenarians and even centenarians running and jumping at master athletics competitions, evidencing a healthy-functional aging induced by lifelong training routines. In addition to the health benefits of regular exercise, we also observed elevated levels of circulating nitric oxide (NO) in middle-aged master athletes (Sousa et al., 2019). This is significant, as NO has antibacterial and antiviral properties that are effective against hepatitis virus and, more to the point for the present time, was effective against the coronavirus *in vitro*, during African Green monkey cells infection (Keyaerts et al., 2004). Given this observation and that previous research has used inhaled NO to treat acute respiratory syndrome (Chen et al., 2004), NO could diminish the complications of a COVID-19 infection. These benefits of higher NO bioavailability could be mediated by enhancing vasodilation, reduction of edema in the alveoli, its antithrombotic effects and inhibition of both neutrophils activation and cytokine release (Green, 2020; Kobayashi and Murata, 2020; Martel et al., 2020). In addition, NO interferes with the interaction between COVID-19 and the ACE-2 receptor via S-nitrosylation of viral proteins (Green, 2020).

Back to the master athletes, their elevated NO, together with their longer leukocyte telomere length, could well-benefit their innate immunity and during an infection limit the associated pulmonary complications, as proposed in Figure 1.

## Physical Fitness, Lifestyle, and Daily Recommendations

During this COVID-19 pandemic, it has been suggested that physical fitness may help risk-stratifying patients (Ahmed, 2020). The publication even proposed COVID-19-specific recommendations of exercise to boost the physiological defense systems “precondition” to combat an infection. It should be noted, however, that the better health of master athletes is not just the result of lifelong high-intensity training routines only (Kusy and Zielinski, 2015), but also resulting from a healthy lifestyle, proper periods of rest/sleep, good stress management and a healthy diet (Korhonen et al., 2014), not even counting psychological traits enhancement. In this regard, we postulate that athletic training-improved motivation, discipline, determination, and resilience are indeed critical to face any kind of diseases or treatments with success.

Although we clearly promote that maintaining physical activity is crucial, we also have some word of caution. Acute, particularly high-volume intensive exercise sessions can cause transient immunosuppression (i.e., 1–3 h post-exercise; Kakanis et al., 2010). Therefore, we recommend that during this pandemic, training sessions should be of moderate-intensity aerobic exercise (<80% maximum heart rate reserve) up to 60 min duration to prevent immunessupression (Kakanis et al., 2010). For resistance exercise, no such limitation is needed as no negative impacts of these intensities in post-exercise immunity have been observed in the elderly (da Cunha Neves Jr et al., 2009).

A transient exercise-induced immunosuppression described above may result in a transient post-exercise “open-window” of diminished ability to combat an infection, and during that period some situations representing an increased risk (i.e., going to supermarket) should be avoided. Nevertheless, in times as these of a pandemic and social isolation, it is important to avoid deconditioning as it will weaken the immune function and diminish the defense against viral infections (Agha et al., 2020). So, protect yourself, be healthy and keep exercising at moderate intensity for your health.

## Conclusion

The higher physical fitness, better health, lower inflammation, better redox balance, and the longer leukocyte telomere length of master athletes indicates they are not only biologically younger than their calendar age suggests, but are also expected to have a better ability to successfully face a COVID-19 disease than their frail sedentary age-matched peers (Figure 1).

## Author Contributions

HS proposed the subject. HS, TR, CS, SA, MK, HD, and CC discussed the hypothesis and the manuscript content. HS wrote the first draft, with CS, SA, MK, and HD making initial revisions. All final revisions were shared and had contributions of HS, CC, DM-S, SA, TR, MK, and HD. All authors read and approved the final manuscript.

## Conflict of Interest

The authors declare that the research was conducted in the absence of any commercial or financial relationships that could be construed as a potential conflict of interest.
